# Influence of Body Composition on Physical Literacy in Spanish Children

**DOI:** 10.3390/biology10060482

**Published:** 2021-05-29

**Authors:** María Mendoza-Muñoz, Sabina Barrios-Fernández, José Carmelo Adsuar, Raquel Pastor-Cisneros, María Risco-Gil, Miguel Ángel García-Gordillo, Jorge Carlos-Vivas

**Affiliations:** 1 Health, Economy, Motricity and Education Research Group (HEME), Faculty of Sport Sciences, University of Extremadura, 10003 Cáceres, Spain; mamendozam@unex.es (M.M.-M.); jadssal@unex.es (J.C.A.); raquelpc@unex.es (R.P.-C.); mariariscogil@gmail.com (M.R.-G.); jorge.carlosvivas@gmail.com (J.C.-V.); 2 Social Impact and Innovation in Health (InHEALTH) Research Group, Faculty of Sport Sciences, University of Extremadura, 10003 Cáceres, Spain; 3 Facultad de Administración y Negocios, Universidad Autónoma de Chile, Sede Talca 3467987, Chile; miguel.garcia@uautonoma.cl

**Keywords:** physical literacy, exercise, health-related quality of life, paediatric obesity, body composition, body mass index

## Abstract

**Simple Summary:**

Childhood overweight and obesity constitute one of the primary public health problems that need to be addressed in advanced societies. The Canadian Assessment of Physical Literacy (CAPL) constitutes one of the most significant initiatives to prevent and combat the issue of childhood obesity in Canada. This assessment is one of the most closely aligned with the concept of a child’s physical literacy and could be a broader alternative to the mere assessment of physical fitness as it assesses the multiple aspects that compose it. Despite its important implications, there is a lack of such studies in Spain. Due to this, the present study aims to establish the level of physical literacy in overweight and non-overweight children in Extremadura (Spain), analyse the differences between them and examine the association among body composition and physical literacy domains. The results showed that most of the non-overweight children in Extremadura (Spain) had higher levels of physical literacy than their overweight counterparts who present lower performance. Furthermore, physical literacy of overweight children was significantly lower compared to non-overweight children. Normal range of body composition values were associated with better total physical literacy and their domains, so a better physical literacy is associated with healthier body composition values.

**Abstract:**

Background: Childhood obesity is a major health challenge in modern societies; therefore, prevention and management policies are needed. This study aims to establish the level of Physical Literacy (PL) in overweight and non-overweight children in Extremadura (Spain), analysing the potential differences between them and exploring their relationships with body composition. Methods: A single-measure cross-sectional study was performed with 135 children, who were taken anthropometric measurements and administered The Canadian Assessment of PL Development (CAPL-2) to assess their level of PL. The CAPL-2 covers four domains and participants to be classified into four levels: beginning, progressing, achieving, and excelling. Statistical analysis included descriptive and correlations. Results: Significant differences between overweight and non-overweight participants were found. Non-overweight participants had higher scores in all the domains included in PL, with the PL level of overweight children mainly being in the two lowest levels. Inverse correlations between body composition variables and PL in all domains (*r* from −0.223 to −0.507) were found, except for the knowledge domain. Conclusions: Most of the non-overweight children had higher levels of PL than their overweight counterparts. The PL of overweight children was significantly lower compared to non-overweight children. Healthier body composition values were associated with a better PL.

## 1. Introduction

Childhood overweight and obesity, defined as an excessive accumulation of body fat, represent one of the major public health issues that need to be addressed in advanced societies [1]. Specifically, Spain presents one of the highest rates in the world [2]. Multiple factors influence the development of overweight and obesity (genetic, neuroendocrine, related to energy expenditure or environmental factors). Specifically, energy expenditure and environmental factors are two of the most relevant in terms of modifying harmful behaviours, such as a sedentary lifestyle, considered as the disease of the 21st century [3,4]. Thus, effective obesity control practices require handling physiological concepts including body composition. Body Mass Index (BMI) is the most widely used anthropometric method to classify overweight and obesity, which is the result of dividing weight in kilograms by height squared in meters [5]. Although there is no consensus on the parameters to consider overweight or obesity based on BMI [6], the Child Growth Standards from the World Health Organization (WHO) [7], followed by the Childhood Obesity Surveillance Initiative (COSI) [2] and others, are generally used.

Physical Literacy (PL) is well-defined as the physical competence, motivation, confidence, knowledge and understanding to participate in physically active lifestyles, and is formed by four domains: physical, psychological, social and cognitive [8,9,10]. PL assumes that the development of essential movement and sportive skills will stimulate the acquisition of active and healthy behaviours, so a physically literate child will skilfully and confidently move in a variety of physically challenging situations, being able to respond intelligently in a variety of physical environments [8,10]. In contrast, a child who has not yet developed a high level of PL will avoid physical activity whenever possible, due to their minimal confidence in their physical ability and their lack of motivation to participate in structured physical activity [11]. According to this, the PL could explain children’s participation or non-participation in physical activities [10], acting as a warning indicator for the need to implement strategies to help them to lead a more active life [12].

The Canadian Assessment of Physical Literacy (CAPL) constitutes one of the most significant initiatives to prevent and combat the issue of childhood obesity in Canada [13]. This assessment is one of the most closely aligned with the concept of a child’s PL and could be a broader alternative to the mere assessment of physical fitness, as it assesses the multiple aspects that compose it.

Despite its important implications, to the best of our knowledge, there is a lack of such studies in Spain [14]. Few studies have evaluated separately how the different domains that form PL influence overweight and obese children’s health: they perform fewer daily behaviours related to physical activity [15], have lower physical condition [16] and reduced motivation towards physical activity [17]. Due to the relevance of studying all the PL domains together, this study aims to (1) establish the level of PL in overweight and non-overweight children in Extremadura (Spain), (2) analyse the potential differences between them and (3) explore the relationship between body composition and the PL domains.

## 2. Materials and Methods

### 2.1. Sample Size

A total of 72 participants were needed to reach a power of 95% to detect a difference of 0.31 between the null hypothesis correlation of 0.29 (very low or close to zero association) and the alternative hypothesis correlation of 0.60 (high association) [18]. The significance level was set at alpha equal to 0.05.

### 2.2. Participants

A single-measure cross-sectional study was carried out with 135 children aged between 8 and 12 years (not overweight: 83 (61.5%); overweight: 52 (38.5%), 63 (46.7%) being male and 72 (53.3%) female. Participants were recruited through different educational centres in Extremadura (Spain).

Participants met the following eligibility criteria: (1) aged 8–12 years; (2) authorised by their parents or legal guardians; (3) children agreed to participate in the study; (4) not having pathologies that prevent participation in physical fitness tests or practice.

### 2.3. Ethics

The Bioethics and Biosafety Committee of the University of Extremadura approved the study (approval number: 23/2021), following the Helsinki Declaration updates, modified by the 64th General Assembly of the World Medical Association (Fortaleza, Brazil, 2013) and the Law 14/2007 on Biomedical Research.

### 2.4. Procedures and Measures

Several tools were utilized to assess PL and body composition of participants. Procedures were conducted according to the study protocol WOMO [19], which allowed monitored outcomes associated to overweight and obesity and the children’s and adolescents’ lifestyles. All participants could practice the physical tests before conducting any measurements.

#### 2.4.1. Anthropometrics

The measurements were conducted under standardized conditions, according to the WHO guidelines [7], previously followed by COSI [2] and ALADINO reports [20]. Participants were asked to remove their shoes and socks, heavy clothing (coats, jumpers, jackets, etc.) and accessories (headbands, pendants, etc.).

Height was determined using a height rod (Tanita Tantois, Tanita Corporation, Tokyo, Japan) located on a vertical surface perpendicular to the ground, in cm, up to the nearest mm. It was assessed standing up, with balanced shoulders and relaxed arms along the body.

Bodyweight and FM were measured with a bioimpedance meter (Tanita MC-780 MA, Tanita Corporation, Tokyo, Japan), as bioelectrical impedance analysis (BIA) technology has been used in studies offering high reproducibility in assessing both FM (ICC = 0.88) and %FM (ICC = 0.66) [21]. Moreover, BIA and DEXA have displayed high correlations for %FM (r = 0.852, ICC = 0.84, concordance coefficient = 0.844) and FFM (r = 0.976, ICC = 0.95, concordance coefficient = 0.955) in youth independent of their physical activity level [22]. The assessment was performed using the “standard mode”, introducing participants’ sex, age and height. Bodyweight was recorded in kg, up to the nearest 100 g. BIA was also used to determine FM and FFM, following the recommendations for a proper measurement [23,24]: more than 3 h since the individual wakes up and since the last intake, not having eaten or exercised heavily during the previous 12 h and urinating before the measurement.

#### 2.4.2. Physical Literacy

**Figure 1:**
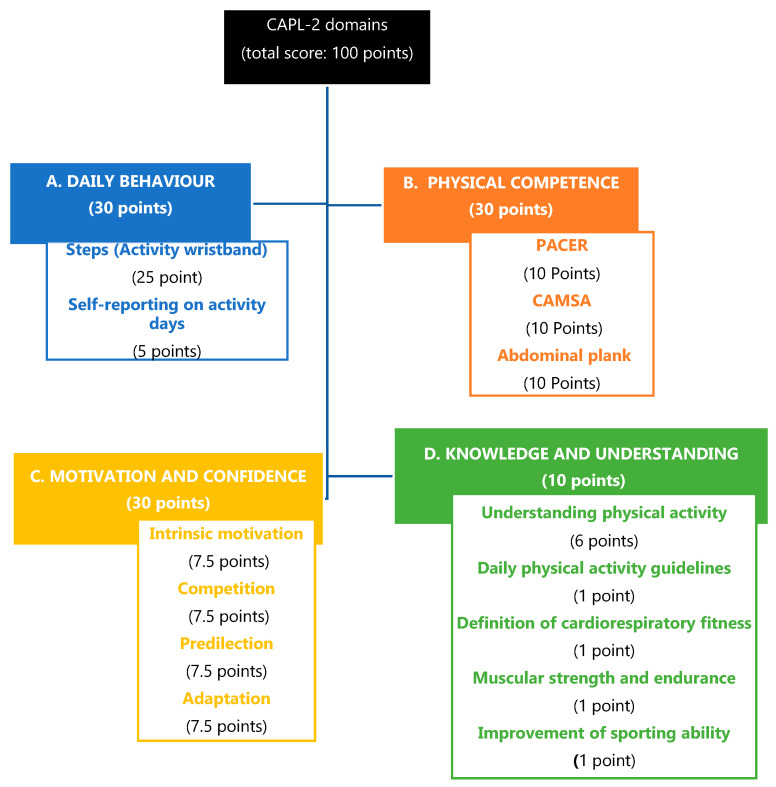
The Canadian Assessment of PL Development (CAPL-2) domains, assessment tests and scoring system, adapted from Longmuir et al. (2018) [28]. CAPL-2: The Canadian Assessment of PL Development; CAMSA: Canadian Agility and Movement Skill Assessment; PACER: Progressive Aerobic Cardiovascular Endurance Run.

The Canadian Assessment of PL Development (CAPL-2) was applied [11,25]. This assessment covers 4 domains: daily behaviour, physical competence, motivation and confidence, knowledge and understanding. Thus, the domains are made up of different tests, which together sum up the score for each domain (Figure 1).

1. Daily behaviour domain. The total score is made up of the scores of 2 components: total steps using an activity wristband (Xiaomi mi Band 3, Xiaomi Corporation, Pekin, China) and a self-administered question about the minutes of physical activity per week performed for at least 60 min.
2. Physical competence domain. The final score is calculated as the sum of 3 components: abdominal plank [26], Progressive Aerobic Cardiovascular Endurance Run (PACER) to determine the cardiorespiratory competence, and the Canadian Agility and Movement Skill Assessment (CAMSA) [27] to measure the participants’ motor skills through an agility circuit. All of them are evaluated with a possible score of 1 to 10 points, totalling among them 1 to 30 points.
3. The motivation and confidence domain assessed confidence and the motivation to be physically active. The scores are obtained by summing 4 parts: intrinsic motivation, competition, predilection and appropriateness, scoring from 1 to 30 points.
4. Knowledge and understanding domain [25]. The score is obtained by answering 5 questions: 4 multiple-choice questions from 0 to 1 point, and one consisting of a fill-in-the-blanks question to complete a story, scoring from 1 to 6. Thus, this domain score can range from 1 to 10.

Thus, the CAPL-2 total score is formed by the sum of the 4 domains scoring from 0 to 100 points. According to their total score, gender and age, the participants are classified into 4 different levels: beginning, progressing, achieving and excelling. The beginning and progressing levels describe children without an optimal level of PL; the achieving one describes children who have met the minimum level and the excelling level describes children who demonstrate a high level of PL [28].

### 2.5. Statistical Analysis

IBM SPSS Statistics 24 software (Armonk, NY: IBM Corporation) was used for statistics. Mean ($\bar{x}$), standard deviation (SD), and median and interquartile range (IR) of data are presented.

Every child’s weight status was established based on the standard deviation of BMI criteria: <−3 SD, “low-weight”; <−2 SD, “normal weight”; >+1 SD, “overweight”; >+2 SD, “obese”. “Low-weight” children were not considered because they accounted for a very low percentage of the sample. Children with “normal weight” were considered in the non-overweight category, and the “overweight” and “obese” categories were taken as a single group named overweight children when conducting study comparisons and computations.

Kolmogorov–Smirnov and Levene’s tests were applied to check normality and homogeneity of data, respectively. Independent Student’s *t*-tests were applied to establish sex differences for parametric variables (height and PL total score). Likewise, Mann–Whitney U-test was used with non-parametric variables (weight, BMI, FM, %FM and CAPL-2 all domains score). Differences were considered significant for *p* ≤ 0.05. To quantify the association among the different variables, Pearson’s (parametric variables) and Spearman’s correlation coefficients (non-parametric variables) were applied. After applying Bonferroni post-hoc correction [29], the alpha significance level was set at 0.012 for multiple comparisons between body composition and PL variables. Correlation values were interpreted following Cohen’s classification thresholds [18]: 0.30 to 0.59, moderate; 0.60 to 0.79, high; ≥ 0.80, excellent.

## 3. Results

Significant differences between overweight and non-overweight participants were found. Specifically, non-overweight participants had higher scores in all the PL domains, in its total score, and lower in weight, height, BMI, FM and %FM (see Table 1).

The PL level of overweight children is mainly in the two lowest levels, beginning and progressing. However, their non-overweight counterparts are mostly placed at the excelling level (42.2%), showing the same level in physical competence (38.6%) and motivation (41%). Both overweight and non-overweight children are mostly at the progressing level in the daily behaviour and knowledge domains (Table 2).

Lastly, correlations between body composition and PL domains and PL total score are shown in Table 3. An inverse correlation between body composition variables and PL in all domains (r from −0.223 to −0.507) was found, except for the knowledge domain.

## 4. Discussion

This study aimed to establish the PL level in overweight and non-overweight children in Extremadura to investigate possible differences between them and to analyse the association between body composition and PL. We found a lower level of PL in overweight compared to non-overweight children. Therefore, significant differences were established between them for all domains of the CAPL-2. In addition, this study displays the existence of a slight to moderate inverse correlation between body composition and all the domains of PL, except in the knowledge domain. These results are relevant and support the establishment of measures for the prevention and management of obesity in children and adolescents.

Related to their level of PL, 63.9% of non-overweight children were in the highest levels (achieving and excelling), while 71.2% of overweight children were in the lowest levels (beginning and progressing). Compared to previous studies, a higher percentage of non-overweight children reached higher levels of PL. In a study conducted in Canada [30], only 44% reached sufficient and excellent levels. Another study carried out in Greece [31] also placed more than 50% of their students in the initial levels (beginning and progressing). A reason should be that both studies did not differentiate by weight category, so it can be argued that non-overweight children can achieve a higher level of PL. Nevertheless, for the daily activity domain, few non-overweight and overweight children reached the achieving and excelling levels. It is a relevant aspect since it confirms the incidence of sedentary lifestyles in children [3,4]. Our findings showed that more than 50% of children did not reach an appropriate level in the daily activity domain. Currently, the WHO recommendations for children aged among 5 and 17 years include performing at least 60 min of moderate to vigorous physical activity per day. In Spain, the PASOS study [32] revelated that only 36.7% of children and young people met these recommendations.

Regarding the differences between overweight and non-overweight children on PL and its domains, it was found that the total score mean on the CAPL-2 for non-overweight children was 71.7 points. This score is significantly higher than that obtained for their overweight counterparts, with an average of 60.76. Our results also agree with those reported by Nystrom et al. [12], who also found significant differences in favour of non-overweight children in their total score (62.8 versus 59.3 points) and for each domain.

Significant differences between overweight and non-overweight children have been previously reported in several studies for the different domains. Concerning daily activity, it has been found that obese children perform less daily activity related to physical activity than non-obese children [15,33,34]. Related to physical competence, worse physical competence was shown in overweight children compared to those of normal weight [35,36,37,38,39], as well as for motivation towards physical activity, which is also lower in overweight children [17]. Regarding the level of knowledge of physical activity, few studies have checked this factor in isolation between overweight and obese children but Nyström, Traversy, Barnes, Chaput, Longmuir and Tremblay [12] reported greater knowledge in favour of non-overweight children.

Furthermore, our study observed an inverse correlation between FM and %FM with PL and its domains. Specifically, higher BMI, FM and %FM values were associated with lower PL total score and every domain score. Nystrom et al. [12] noted that healthy weight children presented higher CAPL scores than their overweight peers. Moreover, Caldwell, et al. [40], using the Physical Literacy Assessment for Youth (PLAY), found a negative association between the %FM and PL, health-related quality of life and blood pressure.

Regarding the knowledge domain, no correlation was obtained with body composition variables except for BMI (very low correlation; *r* = −0.191). It could be because although knowledge is an important element for physical activity promotion, it may not be sufficient to change this behaviour [41]. Furthermore, the relationship between the knowledge domain and the other domains was the lowest [12]. Thus, significant differences in favour of non-overweight children could be explained or associated with higher levels of physical competence that could indirectly be a source of knowledge.

The present study has different limitations that should be considered in future research. All participants were from the autonomous community of Extremadura (Spain), so cultural differences may influence the generalisation of results. Moreover, it was not possible to establish causal relationships among PL and body composition due to the cross-sectional design of this study.

## 5. Conclusions

Most of the non-overweight children in Extremadura (Spain) have higher levels of PL (sufficient and excellent) than their overweight counterparts, which presents lower performance (insufficient and in progress). Furthermore, PL of overweight children was significantly lower compared to non-overweight children. A normal range of body composition values were associated with better total PL and their domains, so a better PL is associated with healthier body composition values.

## Figures and Tables

**Table 1:** Anthropometry and PL of overweight and non-overweight participants.

	Total		Non-OW		OW		*p*
N (%)	135 (100)		83 (61.5)		52 (38.5)		
	Median	IR	Median	IR	Median	IR	
**Age (years)**	11.00	2.00	11.00	2.00	11.00	2.00	0.19
**Bodyweight (kg)**	39.10	13.60	34.50	8.60	48.60	14.65	<0.001 *
**BMI (kg/m^2^)**	18.50	5.00	16.20	3.30	21.45	3.95	<0.001 *
**Fat Mass (kg)**	8.80	6.10	6.50	3.70	12.75	7.08	<0.001 *
**% Fat Mass**	22.80	9.20	19.40	6.10	28.20	7.98	<0.001 *
**Physical activity domain (points)**	18.00	11.00	20.00	10.00	15.00	10.25	0.002 *
**Physical competence (points)**	18.00	11.71	20.86	9.50	14.36	10.89	<0.001 *
**Motivation and confidence domain (points)**	24.60	4.00	24.80	4.70	23.49	3.63	0.022 *
**Knowledge and understanding domain (points)**	7.00	2.00	7.00	2.00	7.00	3.00	0.035 *
	**Average**	**SD**	**Average**	**SD**	**Average**	**SD**	
**Height (cm)**	146.15	9.23	144.58	8.84	148.67	9.38	0.012 *
**Total PL score (points)**	67.49	13.58	71.70	12.37	60.76	12.79	<0.001 *

OW: overweight children; Non-OW: non-overweight children. Daily behaviour domain, physical competence and motivation and confidence domain scores are 1 to 30 points each; knowledge domain is 1 to 10 points; total PL score is 1 to 100 points. The *p*-value represents the results of Student’s *t*-tests (parametric variables) and Mann–Whitney U-test (non-parametric variables) to establish differences between weight categories; * *p* < 0.05.

**Table 2:** Overweight and non-overweight children at the 4 levels of CAPL-2 [28] and their domain scores, total and percentages.

		Beginning	Progressing	Achieving	Excelling
**Daily behaviour**	OW	11 (21.2)	29 (55.8)	6 (11.1)	6 (11.1)
	Non-OW	6 (7.2)	42 (50.6)	19 (22.9)	16 (19.3)
**Physical competence**	OW	27 (51.9)	15 (28.8)	3 (5.8)	7 (13.5)
	Non-OW	17 (20.5)	22 (26.5)	12 (14.5)	32 (38.6)
**Motivation and confidence**	OW	-	25 (48.1)	16 (30.8)	11 (21.2)
	Non-OW	-	24 (28.9)	25 (30.1)	34 (41.0)
**Knowledge and understanding**	OW	12 (23.1)	25 (48.1)	9 (17.3)	6 (11.5)
	Non-OW	10 (12.0)	36 (43.4)	20 (24.1)	17 (20.5)
**Total PL score**	OW	11 (21.2)	26 (50.0)	8 (15.4)	7 (13.5)
	Non-OW	5 (6.9)	25 (30.1)	18 (21.7)	35 (42.2)

OW: overweight children; Non-OW: non-overweight children.

**Table 3:** Correlations between body composition and physical literacy and its domains.

	BMI	FM	%FM
**Daily behaviour domain**	−0.261 **	−0.266 **	−0.272 **
**Physical competence domain**	−0.433 **	−0.471 **	−0.507 **
**Motivation and confidence domain**	−0.223 **	−0.268 **	−0.241 **
**Knowledge and understanding domain**	−0.191 *	−0.139	−0.152
**Total PL score**	−0.446 **	−0.478 **	−0.491 **

* Significance level 0.05; ** Significance level 0.012. BMI: Body Mass Index; FM: fat body mass; %FM: percentage of fat body mass.

## Data Availability

The datasets used during the current study are available from the corresponding author on reasonable request.
